# Supplementary material for: Self-generation and sound intensity interactively modulate perceptual bias, but not perceptual sensitivity
Source: Sci Rep. 2021 Aug 24;11:17103. doi: 10.1038/s41598-021-96346-z (PMC8385100; doi:10.1038/s41598-021-96346-z)
Supplement: Supplementary file 1 — Supplementary Information. [file 41598_2021_96346_MOESM1_ESM.pdf]

Self-generation and sound intensity interactively modulate perceptual bias, but not perceptual sensitivity

Nadia Paraskevoudi[a,b] & Iria SanMiguel[a,b,c*]

Affiliations:

[a] Brainlab-Cognitive Neuroscience Research Group, Department of Clinical Psychology and Psychobiology, University of Barcelona, Barcelona, Spain
[b] Institute of Neurosciences, University of Barcelona, Barcelona, Spain
[c] Institut de Recerca Sant Joan de Déu, Esplugues de Llobregat, Spain

**Corresponding author:*

Iria SanMiguel
Department of Clinical Psychology and Psychobiology
P. Vall d'Hebron 171
08035 Barcelona, Spain
Tel: (0034) 93 312 5050
Email: isanmiguel@ub.edu

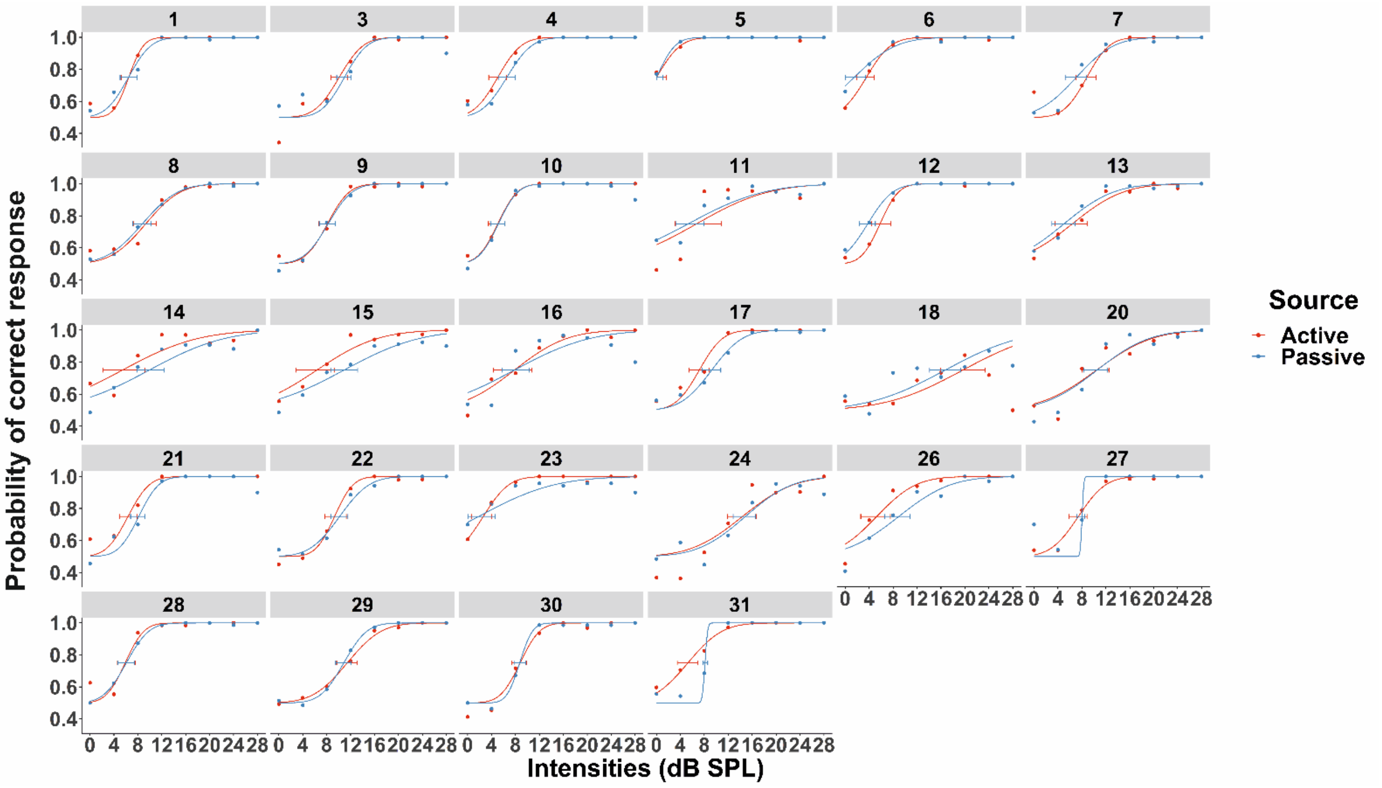

Figure S1. Psychometric functions for 28 participants from the detection task fitted to the percent correct responses as a function of sound intensity. Number in the legend above each plot corresponds to each participant's number (participants with numbers 2, 19, 25 were excluded; see Methods). The small horizontal segments represent the 95% confidence intervals for thresholds (parametric bootstrap procedure with n = 1000). The threshold is defined as the intensity accurately detected at 75% of the trials (as derived from the psychometric function fitted for each participant) and is represented by the intersection of the confidence interval with the psychometric function.

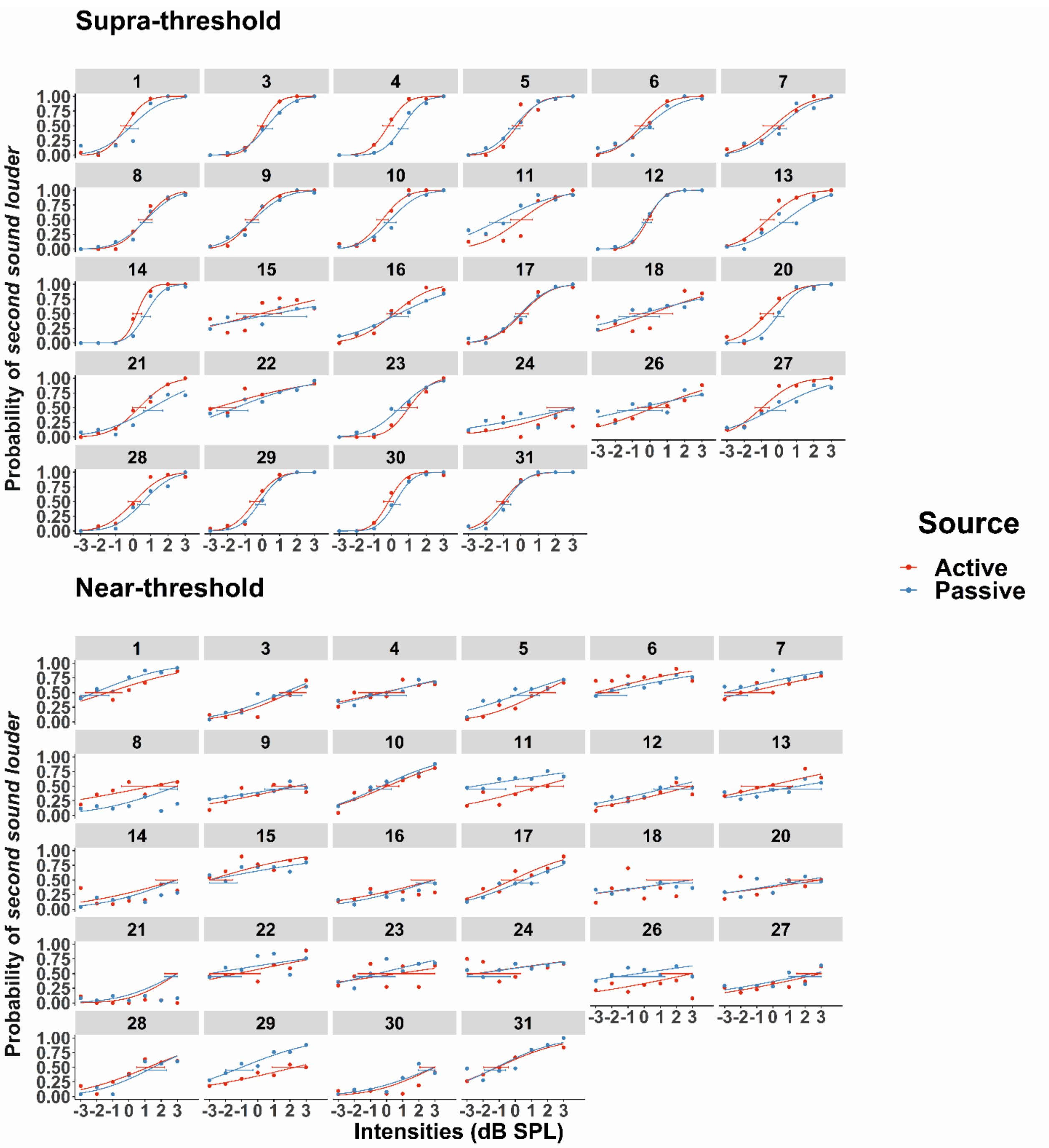

Figure S2. Psychometric functions for 28 participants from the discrimination task fitted to the probability of judging the comparison sound as louder as a function of its difference in dB from the first standard tone (±3 dB in steps of 1) for the supra- and the near-threshold intensities, respectively. Number in the legend above each plot corresponds to each participant's number (participants with numbers 2, 19, 25 were excluded; see Methods). The small horizontal segments represent the 95% confidence intervals (parametric bootstrap procedure with n = 1000) for the point of subjective equality (PSE), which is defined as the intensity, where the comparison stimulus was reported as louder than the standard one on 50% of the trials.

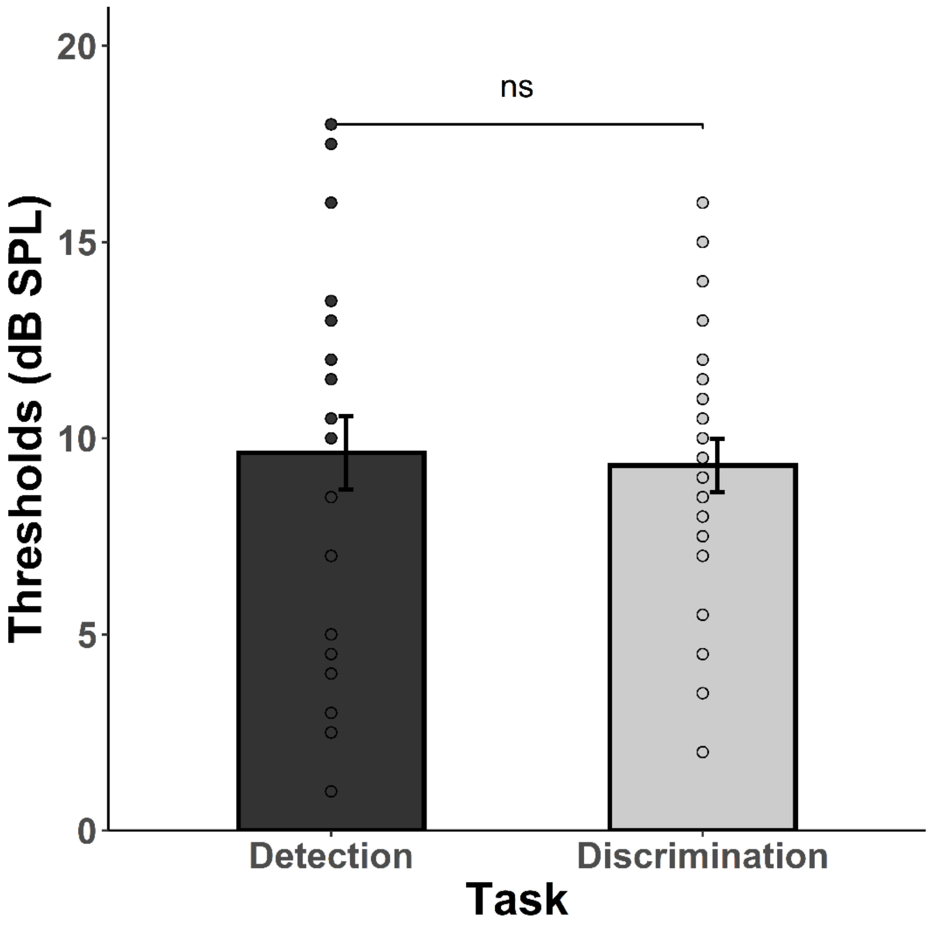

Figure S3. Mean audiometric thresholds prior to each task. From each audiometry, we obtained the thresholds for both the left and right ear. For all subjects, the thresholds were below 20 dB. Considering that in both tasks, we utilized a pure tone of 1000 Hz, in this analysis we only considered the thresholds for the 1000-Hz sounds. The mean thresholds across ears for each condition were introduced in a statistical analysis using a paired-sampled two-sided t-test to test for differences in audiometric thresholds prior to each task. The analysis did not show any significant differences ($M_{AM\_Detection}$ = 9.63, $M_{AM\_Discrimination}$ = 9.3, $SD_{AM\_Detection}$ = 4.94, $SD_{AM\_Discrimination}$ = 3.59, $p > .050$; Shapiro-Wilk normality test, $p > .050$).

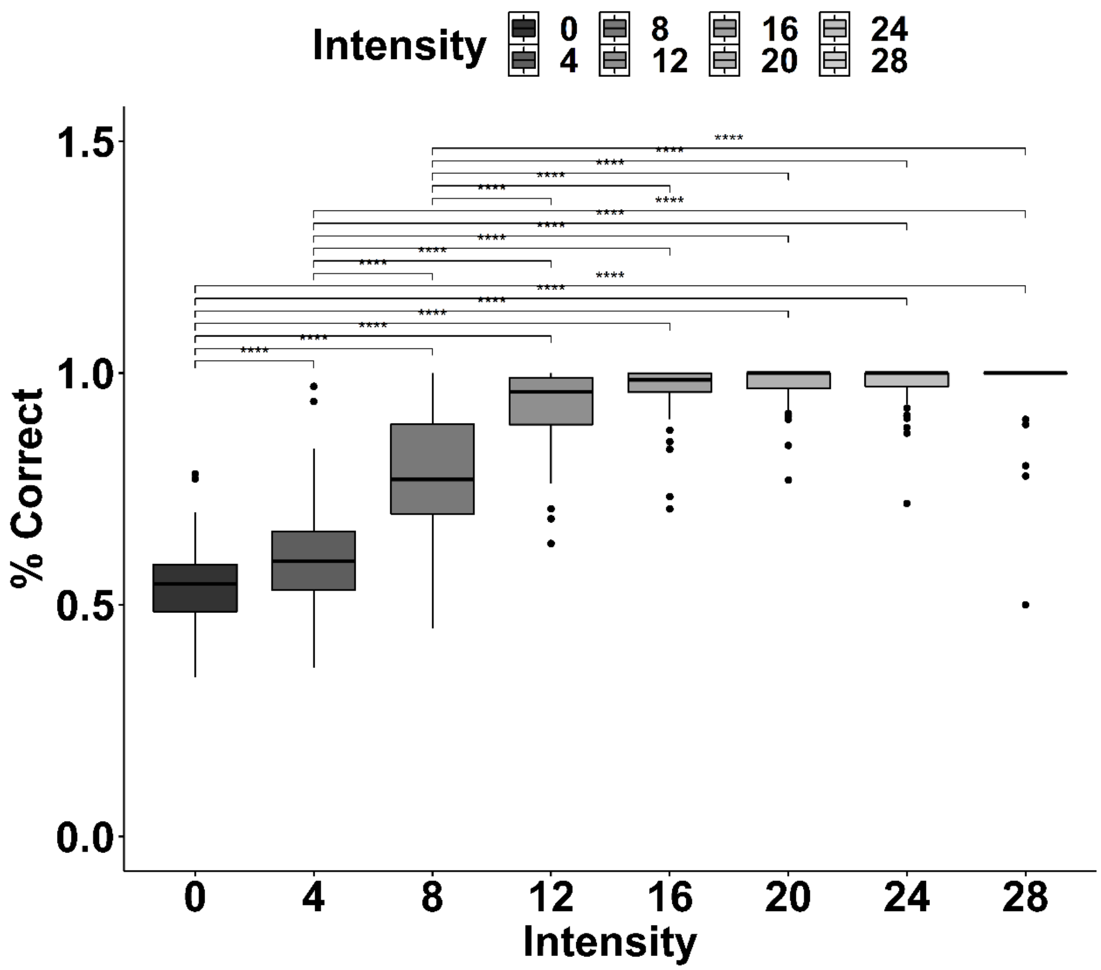

Figure S4. Repeated measures ANOVA with factors Intensity (0, 4, 8, 12, 16, 20, 24, 28) and Source (active and passive) on detection accuracy. The Greenhouse-Geisser correction was applied where sphericity was violated. The analysis did not show any significant main effect of source ($F(1,27) = 1.64$, $p > .050$), but we obtained a significant main effect of intensity, $F(2.35,63.44) = 228.79$, $p < .001$, $\eta_p^2 = .89$ and $\eta_G^2 = .78$. Specifically, irrespective of whether the sound was self- or externally-generated, participants' accuracy was significantly lower at 0 dBs compared to the rest of the intensities, at 4 dBs compared to the intensities above 8 dBs, at 8 dBs compared to the intensities above 12 dBs, and at 12 dBs compared to intensities above 16 dBs (all $p < .001$; $M_0 = 54.65$, $SD_0 = 8.85$, $M_4 = 61.19$, $SD_4 = 11.9$, $M_8 = 77.99$, $SD_8 = 13.33$, $M_{12} = 92.53$, $SD_{12} = 8.88$, $M_{16} = 96.47$, $SD_{16} = 6.03$, $M_{20} = 97.4$, $SD_{20} = 4.36$, $M_{24} = 97.37$, $SD_{24} = 4.79$, $M_{28} = 97.26$, $SD_{28} = 8.09$). Comparisons between higher intensities (i.e., 16 – 28 dBs) did not show any significant differences in participants' accuracy. The interaction between source and intensity did not reach significance ($F(4.10,110.68) = .62$, $p > .050$).

Table S1. Significant correlation for the values obtained between the two tasks (thresholds/slopes, PSE/JND values)

Correlations between mesures in		***r***	***p***
**Detection**	**Discrimination**		
slope_passive	JND_AN	0.502218293833961	0.006
slope_passive	JND_PS	0.458809161204315	0.014
slope_passive	JND_PN	0.458580361105521	0.014
slope_active	JND_PN	0.456946049747953	0.015
slope_active	JND_PS	0.447376137712047	0.017
slope_active	JND_AN	0.400560869306606	0.035
slope_passive	JND_AS	0.393510389324828	0.038
slope_active	JND_AS	0.37480689145703	0.049

Note. Significant correlations were only obtained between the slopes at the detection task and the JND values at the discrimination task for all conditions (A = Active, P = Passive, S = Supra-threshold, N = Near-threshold). No significant correlations between a) detection thresholds and PSE values, b) slopes at the detection task and PSE values at the discrimination task, c) detection thresholds and JND values at the discrimination task (all $p > .050$).
